# Supplementary material for: Polymer-Degrading Enzymes of Pseudomonas chloroaphis PA23 Display Broad Substrate Preferences
Source: Int J Mol Sci. 2023 Feb 24;24(5):4501. doi: 10.3390/ijms24054501 (PMC10003648; doi:10.3390/ijms24054501)
Supplement: Supplementary file 1 [file ijms-2170674-supplementary.pdf › ijms-2170674-supplementary.pdf]

Supplementary Material

Polymer-Degrading Enzymes of *Pseudomonas chloroaphis* PA23 Display Broad Substrate Preferences

Nisha Mohanan [1], Michael C.-H. Wong [2], Nediljko Budisa [2,3] and David B. Levin [1,*]

[1] Department of Biosystems Engineering, University of Manitoba, Winnipeg, MB R3T 5V6, Canada

[2] Department of Chemistry, University of Manitoba, 144 Dysart Rd., Winnipeg, MB R3T 2N2, Canada

[3] Biocatalysis Group, Technical University of Berlin, Müller-Breslau-Str. 10, D-10623 Berlin, Germany

* Correspondence

Prof. David B. Levin, david.levin@umanitoba.ca

Table of contents

Supplementary Figures S1-S3, pages 2 to 4

Supplementary Table S1, pages 5 and 6

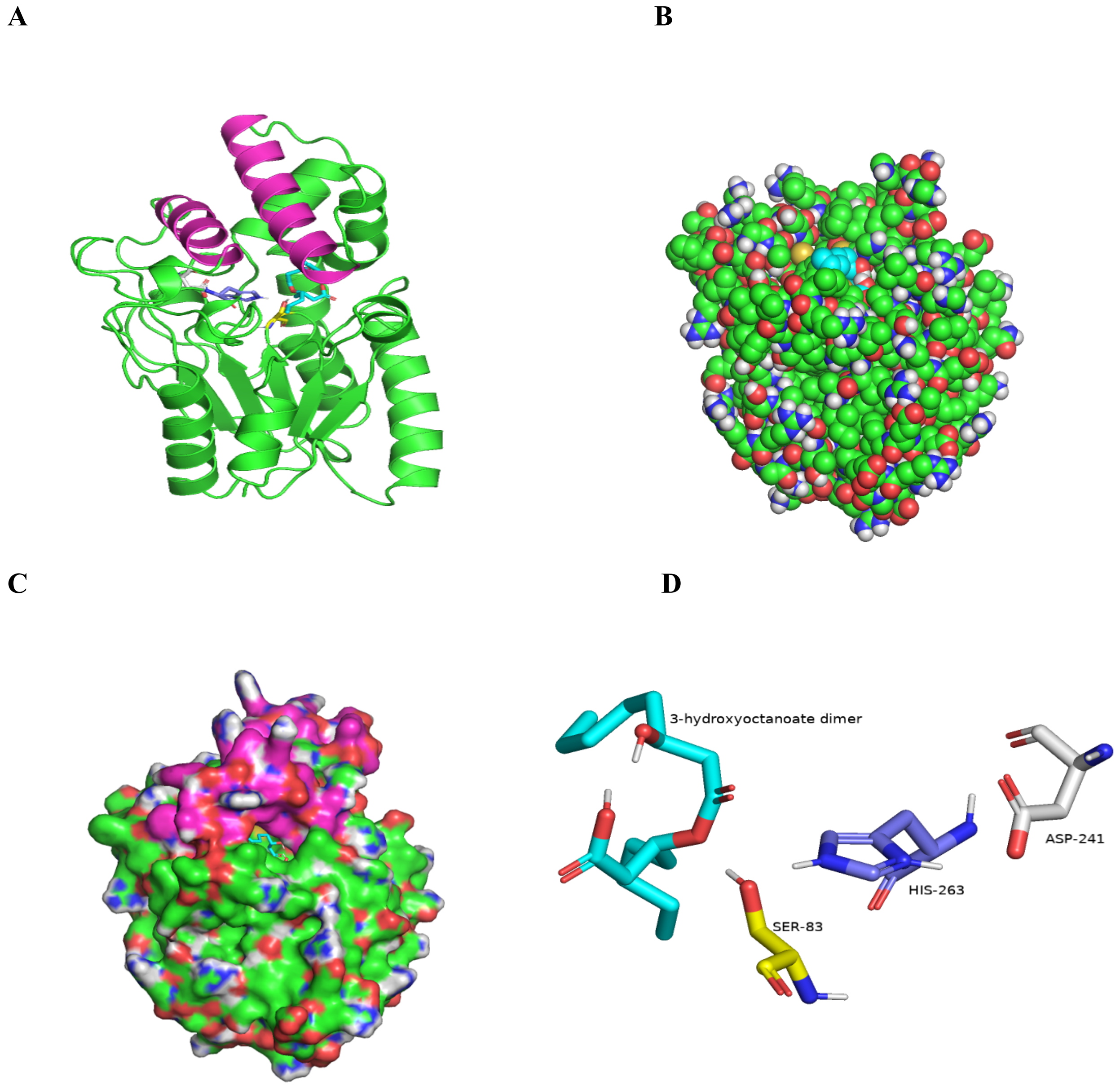

Supplementary Figure S1. Model of the three-dimensional structure of LIP3 using *Proteus mirabilis* lipase as a template. A) General diagram (Ribbon plot) of the LIP3 scaffold with labeled residues of the catalytic triad Ser^{83} (yellow) His^{263} (slate) Asp^{241} (gray) and docked substrate 3-hydroxyoctanoate dimer (cyan), the core domain (green) and the lid domain (magenta). B) Atomic model of LIP3 with docked substrate in van der Waals representation. C) Lipase LIP3 surface representation with the docked substrate (stick representation). D) The residues (stick model) of the catalytic triad around the substrate, colored as in A.

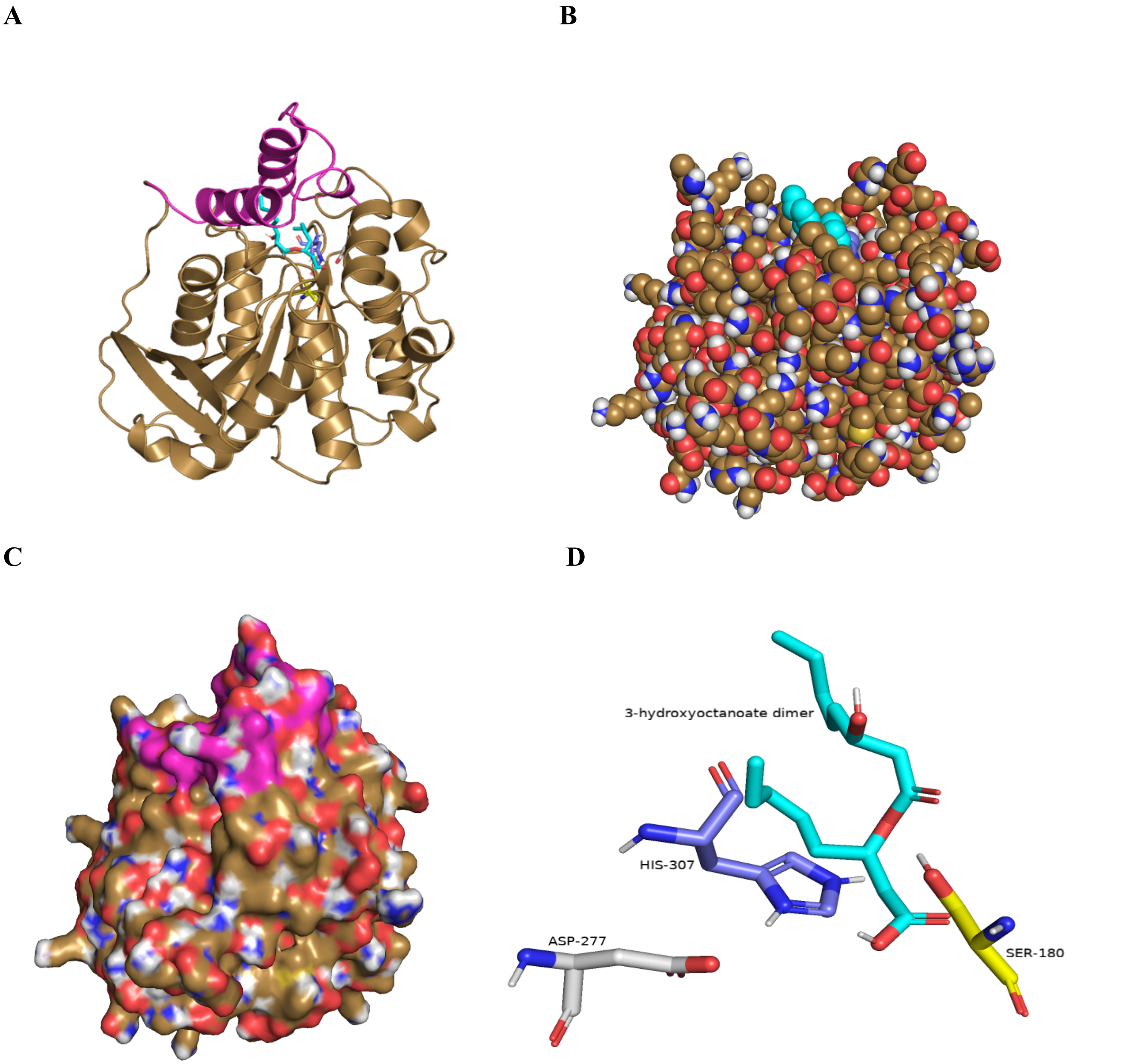

Supplementary Figure S2. Model of the three-dimensional structure of Lip4 using the acetyl esterase CinB from *Enterobacter asburiae* as a template. A) General diagram (Ribbon plot) of the LIP4 scaffold with labeled residues of the catalytic triad Ser^{180} (yellow) His^{307} (slate) Asp^{277} (gray) and docked substrate substrate 3-hydroxyoctanoate dimer (cyan), the core domain (sand) and the lid domain (magenta). B) Atomic model of LIP4 with docked substrate in van der Waals representation. C) Lipase LIP4 surface representation with the docked substrate (stick representation). D) The residues (stick model) of the catalytic triad around the substrate, colored as in A.

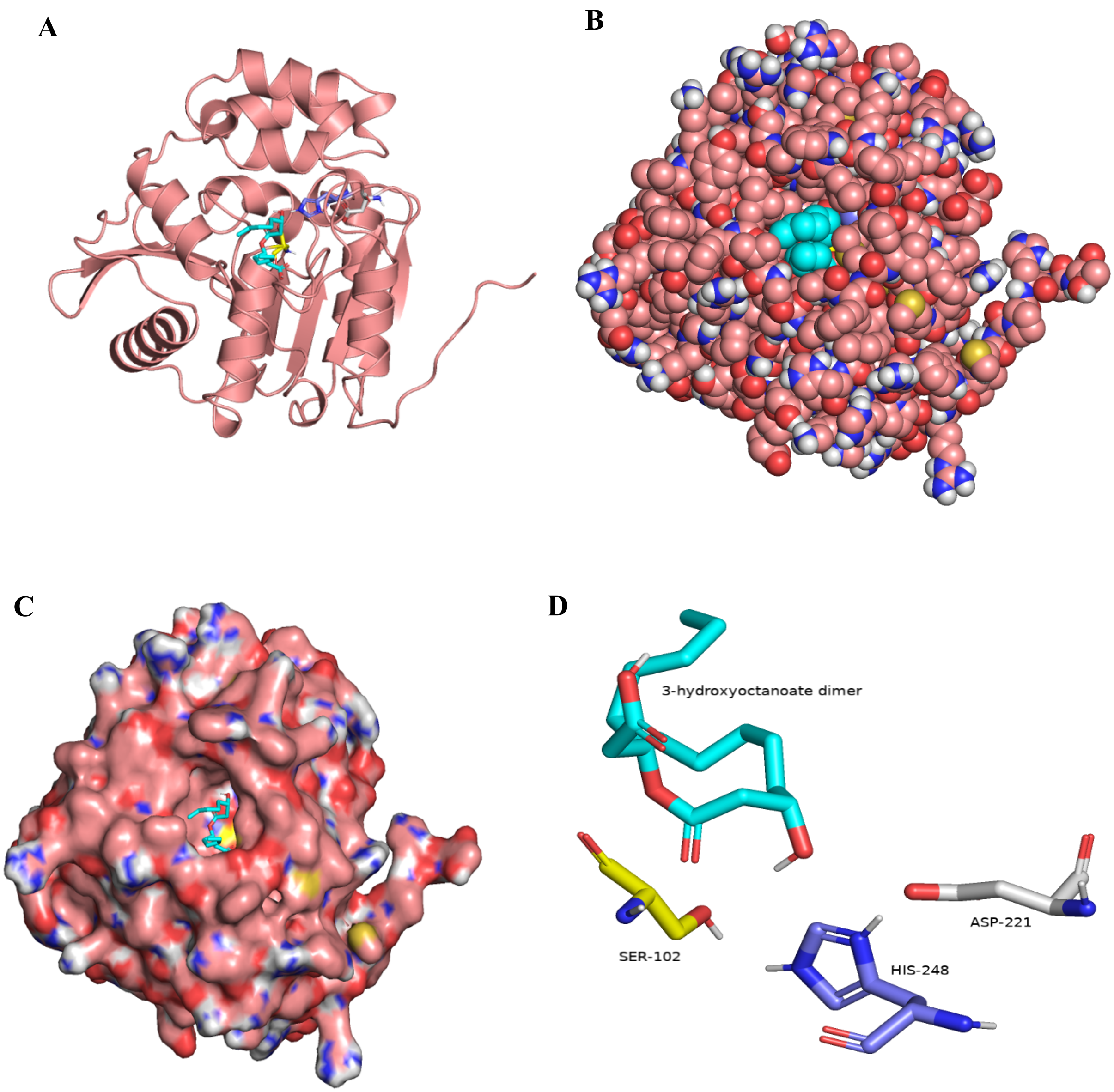

Supplementary Figure S3. Model of the three-dimensional structure of PhaZ using the poly(3-hydroxyalkanoate) depolymerase from *Pseudomonas oleovorans* as a template. A) General diagram (Ribbon plot) of the PhaZ scaffold with labeled residues of the catalytic triad Ser^{102} (*yellow*) His^{248} (*slate*) Asp^{221} (*gray*) and docked substrate substrate 3-hydroxyoctanoate dimer (*cyan*), the core domain (*salmon*) and the lid domain (*magenta*). B) Atomic model of PhaZ with docked substrate in van der Waals representation. C) PhaZ surface representation with the docked substrate (stick representation). D) The residues (stick model) of the catalytic triad around the substrate, colored as in A.

Supplementary Table S1. Quantitative results of GPC analysis showing the changes in polymer properties after treatment with LIP3, LIP4 and PhaZ compared with untreated polymers (control).

Polymer	Test	Peaks	Retention time (Minutes)	Mn (Da)	Mw (Da)	MP (Da)	Mz (Da)	Polydispersity (Mw/ Mn)	% Area	Height
**PHBV**	Control	Peak 1	7.564	23416	42700	34542	70858	1.82	100	19040
	After reaction with LIP3	Peak 1	7.669	18034	34326	28043	56163	1.90	88.13	18638
		Peak 2	9.490			857			11.87	5246
	After reaction with LIP4	Peak 1	7.676	17229	32944	27687	53968	1.91	94.60	19172
		Peak 2	9.448			927			10.14	2023
	After reaction with PhaZ	Peak 1	7.644	19626	37365	29479	65549	1.90	91.99	18034
		Peak 2	9.486			863			8.01	2928
**PHHx**	Control	Peak 1	7.685	21305	42212	27177	80663	1.98	100	18267
	After reaction with LIP3	Peak 1	7.814	14792	28142	20820	48782	1.90	86.73	17337
		Peak 2	9.476			918			13.27	7381
	After reaction with LIP4	Peak 1	7.809	14440	28934	21234	51658	2.00	92.08	19875
		Peak 2	9.432			957			7.92	3577
	After reaction with PhaZ	Peak 1	7.726	16028	33601	23052	63801	1.78	71.73	6050
		Peak 2	9.476			880			28.27	5805
**PHO**	Control	Peak 1	7.803	18931	36805	21477	69468	1.94	100	22399
	After reaction with LIP3	Peak 1	7.961	13714	26373	17157	49023	1.92	88.59	17276
		Peak 2	9.461			905			11.41	4252
	After reaction with LIP4	Peak 1	7.889	14291	27005	18106	48239	1.88	93.74	22672
		Peak 2	9.435			952			6.26	3107
	After reaction with PhaZ	Peak 1	7.793	16385	33489	20146	65639	2.726	75.48	6683
		Peak 2	9.486			862			24.52	8702

**PHN**	Control	Peak 1	7.296	40489	101608	58889	249310	2.51	100	27411
	After reaction with LIP3	Peak 1	7.559	23615	42104	34907	67216	1.78	90.43	18559
		Peak 2	9.468			893			9.57	4039
	After reaction with LIP4	Peak 1	7.381	32890	57596	49711	90088	1.75	87.55	14330
		Peak 2	9.409			1000			12.45	4476
	After reaction with PhaZ	Peak 1	7.374	32974	57424	50418	92513	1.61	100	7994
**PHD**	Control	Peak 1	7.679	17584	34145	27490	57727	1.94	100.00	26827
	After reaction with LIP3	Peak 1	7.830	11607	24335	20348	42737	2.09	93.20	20428
		Peak 2	9.472			887			6.80	3857
	After reaction with LIP4	Peak 1	7.801	11908	25500	21590	45457	2.14	93.06	25498
		Peak 2	9.429			963			6.94	4147
	After reaction with PhaZ	Peak 1	7.700	10471	29858	24302	54757	1.748	100	9005
**PLA**	Control	Peak 1	6.698	111284	209736	191677	329504	1.88	100	23760
	After reaction with LIP3	Peak 1	6.827	88291	169186	148392	269869	1.92	100	28038
	After reaction with LIP4	Peak 1	6.744	108458	195139	174894	304117	1.79	100	27006
	After reaction with PhaZ	Peak 1	6.780	96247	185429	163042	295997	1.93	100	30163
**PCL**	Control	Peak 1	6.711	130101	210551	186692	306961	1.62	100	72723
	After reaction with LIP3	Peak 1	6.877	59427	142106	134503	244037	2.39	100	24883
	After reaction with LIP4	Peak 1	7.055	46562	108800	94663	190941	2.33	100	47550
	After reaction with PhaZ	Peak 1	6.875	84975	153365	135128	235411	1.80	100	69359
**PES**	Control	Peak 1	8.719	2685	4453	3619	6684	1.66	100	50502
	After reaction with LIP3	Peak 1	8.733	2180	4016	3381	5918	1.84	100	48311
	After reaction with LIP4	Peak 1	8.728	2122	4015	3413	5980	1.89	100	49303
	After reaction with PhaZ	Peak 1	8.750	2206	3949	3283	5823	1.79	100	48468

Da, Daltons; Mn, polymer average mass number; Mw, polymer average weight number; MP, peak average molecular weight; Mz, higher average molecular weight
